# Prevalence and Correlates of Overweight, Obesity and Physical Activity in Italian Children and Adolescents from Lombardy, Italy

**DOI:** 10.3390/nu14112258

**Published:** 2022-05-28

**Authors:** Chiara Stival, Alessandra Lugo, Lavinia Barone, Giovanni Fattore, Anna Odone, Silvia Salvatore, Eugenio Santoro, Silvia Scaglioni, Piet A. van den Brandt, Silvano Gallus, HBSC Lombardy Committee, OKKio alla Salute Lombardy Committee

**Affiliations:** 1 Depatment of Environmental Health Sciences, Istituto di Ricerche Farmacologiche Mario Negri IRCCS, 20156 Milan, Italy; chiara.stival@marionegri.it (C.S.); alessandra.lugo@marionegri.it (A.L.); silvano.gallus@marionegri.it (S.G.); 2 Department of Brain and Behavioral Sciences, University of Pavia, 27100 Pavia, Italy; lavinia.barone@unipv.it; 3 Department of Social and Political Sciences & CERGAS-SDA, Bocconi University, 20136 Milan, Italy; giovanni.fattore@unibocconi.it; 4 Department of Public Health, Experimental and Forensic Medicine, University of Pavia, 27100 Pavia, Italy; anna.odone@unipv.it; 5 School of Medicine, University Vita-Salute San Raffaele, 20132 Milan, Italy; 6 Department of Pediatrics, Ospedale “F. Del Ponte”, University of Insubria, 21100 Varese, Italy; silvia.salvatore@uninsubria.it; 7 Laboratory of Medical Informatics, Department of Public Health, Istituto di Ricerche Farmacologiche Mario Negri IRCCS, 20156 Milan, Italy; eugenio.santoro@marionegri.it; 8 De Marchi Foundation—Department of Pediatrics, IRCCS Ospedale Maggiore Policlinico, 20122 Milan, Italy; 9 Department of Epidemiology, Maastricht University Medical Centre, GROW—School for Oncology and Developmental Biology, 6200 MD Maastricht, The Netherlands; pa.vandenbrandt@maastrichtuniversity.nl; 10 Department of Epidemiology, Maastricht University Medical Centre, CAPHRI—School for Public Health and Primary Care, 6200 MD Maastricht, The Netherlands

**Keywords:** childhood obesity, childhood overweight, physical activity, cross sectional study, adolescents, screen time, Italy, HBSC

## Abstract

Investigating pediatric overweight and physical activity correlates is essential to design effective preventive programs. We used regional data (Lombardy, northern Italy) from the 2019 survey “OKKio alla Salute” (3093 children aged 8–9 years with measured anthropometric data), and from the 2018 wave of the “Health Behaviour in School-aged Children” survey (2916 adolescents aged 11–15 years with self-reported anthropometric data). In both the surveys, a cluster sampling methodology was used. Unconditional multiple logistic regression models were applied to estimate the odds ratios (OR) and corresponding 95% confidence intervals (CI) of overweight, obesity and poor physical activity. The prevalence of overweight (including obesity) was 22.4% for children aged 8–9 years and 14.4% for adolescents aged 11–15 years. A higher prevalence of overweight was observed among males, children with greater birth weight and those with obese parents. Scant physical activity was higher among females and older adolescents. There was a direct relationship between obesity and increased psychological distress (OR = 2.44; 95% CI: 1.12–5.27) or being victims of bullying (OR = 2.25; 95% CI: 1.17–4.34). Increasing physical activity significantly decreased the frequency of mental health outcomes. Prevention campaigns should be promoted to safeguard childhood physical and psychological wellbeing.

## 1. Introduction

Over the past three decades Europe has undergone a substantial socio-economic transition that has changed its inhabitants’ dietary habits, leading to a more obesogenic environment [1]. The increased availability, access and affordability of energy-high-caloric foods, along with their intense marketing, are recognized examples of the environmental factors that help to explain excess energy intake and subsequent weight gain [2]. As a consequence, in most European countries the prevalence of overweight in children and adolescents has steadily risen since 1980, reaching its peak in 2007–2010 [3,4]. 

In Italy, childhood overweight and obesity show a special pattern. Italy ranks as the fourth country for overweight (including obesity) among children aged 7–9 years, out of 36 European countries [5], but its position in the ranking drops sharply with age. Out of 43 European countries, Italy ranks 4th among children aged 11 years, 11th among children aged 13 years, and 20th among children aged 15 years [6]. This huge reduction is even more marked in adulthood, with Italy ranking as the country with the lowest prevalence of overweight (including obesity) among 30 countries included in the Eurobarometer data [7]. Estimates from the Italian National Institute of Statistics show similar patterns [8].

There is evidence that the prevalence of childhood overweight and obesity is substantially higher in southern than northern Italy [9,10]. This is also true for the adult general population [10]. 

The increasing tendency to a sedentary lifestyle and insufficient physical activity, even in childhood, in most high-income countries contributes to raise this issue [11]. In the last few decades, several studies have already documented that children and adolescents from most high-income countries have become less physically active and more sedentary [12,13]. Screen time behaviors, such as watching TV, playing videogames or using computers for other purposes, such as social media or surfing the internet, have all contributed to the overall increase in sedentary time. Recent estimates show that less than 20% of European children and adolescents meet the World Health Organization (WHO) recommendations on physical activity [14,15]. A WHO report indicated that the prevalence of adolescents (aged 11–15 years) reaching the recommended level of at least 60 min of physical activity per day in Italy (11%) is lower than in several other European countries [16]. In addition, Italian schools tend to devote less time to physical exercise, compared to those from other European countries [15,17]. This issue is becoming more compelling given the increase in sedentary habits acquired with remote learning during the pandemic [18].

Childhood obesity and scant physical activity are strongly related with poor physical health but less is known about their association with mental health [19]. Some studies, however, have already shown that obese children and those with low levels of physical activity have an impaired psychological wellbeing [20,21,22,23]. Moreover, obese children are often victims of bullying and weight stigmatization [24,25,26]. Therefore, these factors need to be considered in body mass index (BMI) surveillance programs and when planning interventions to improve not only physical but also psychological wellbeing. 

Comprehensively investigating which are the characteristics of the overweight and poorly active children and adolescents is essential to design effective prevention programs. The aim of the present study is to provide up-to-date consistent knowledge on the prevalence and correlates of overweight and poor physical activity among Italian children and adolescents, with special focus on familial characteristics, lifestyle habits and psychological wellbeing, taking advantage of two Italian surveillance systems: OKKio alla Salute, on children aged 8–9 years, and Health Behaviour in School-aged Children (HBSC), on adolescents aged 11–15 years. In order to provide estimates on determinants of overweight and poor physical activity not affected by the geographical gradient, we focused our analysis to Lombardy (northern Italy), the most populous region counting one-sixth of the Italian population [27], with a population size comparable to Sweden and Portugal.

## 2. Materials and Methods

We used Lombardy regional data from the 2019 wave of the Italian National Surveillance System OKKio alla Salute, coordinated by the National Institute of Health (Istituto Superiore di Sanità) and from the 2018 wave of the HBSC survey.

OKKio alla Salute is a national nutritional surveillance system, part of the Childhood Obesity Surveillance Initiative (COSI) of the WHO Regional Office for Europe, established in 2007 with the objective of monitoring the nutritional status and lifestyle behaviors among primary school children. The survey involved children in the third grade of primary schools, aged 8–9 years. A cluster sampling method was used, with classes as the sampling unit. Sampling selection was done at the Local Health Unit level (often corresponding to the Province), with a sampling list of third primary classes provided by the corresponding Provincial School Offices [28]. For each school, the probability of having their classes extracted was proportional to the number of students enrolled. The final sample consisted of 166 classes and 3093 children, with anthropometric measurements provided by trained local health staff. 

HBSC is an international multicenter study [29] conducted in more than 40 countries across Europe and North America, in collaboration with the Regional Office for Europe of the WHO with the aim of monitoring the nutritional status and gaining greater insight into determinants of health and wellbeing in adolescents. The HBSC study population comprises school young adolescents aged 11–15 years. The sampling procedure followed international guidelines. Classes were selected according to a systematic sampling method from the complete list of schools provided by the Italian Ministry of Education, University and Research. Cluster sampling was used to select participants, with school classes representing the primary sampling unit. In the Italian HBSC schools from Lombardy were oversampled to ensure sufficient statistical power to obtain robust frequency estimates at a regional level [30]. In all, 2916 students from 188 classes, self-reporting height and weight, were included in the study. 

Details on sampling methods and data collection in the two surveys are reported elsewhere [28,31]. The protocol of the OKKio alla Salute survey was approved by the Institutional Ethical Board of the National Institute of Health (General protocol: ISS AOO-ISS 12/12/2018 0037697). The protocol of the HBSC survey was approved by the Institutional Ethical Board of the National Institute of Health (General protocol: PRE-876/17).

### 2.1. Outcome Measures

Body mass index (BMI) was calculated according to the sex-and-age specific international Cole’s cut-offs proposed by the International Obesity Task Force (IOTF) [32,33]. Underweight corresponded to a BMI value equivalent to a BMI lower than 18.5 kg/m^2^ extrapolated for the 18 years population, the overweight definition corresponded to a BMI value equivalent to a BMI ranging between 25–29 kg/m^2^ extrapolated for the 18 years population and the obesity definition corresponded to a BMI value ≥ 30 kg/m^2^ extrapolated for the 18 years population, for age and sex. Children and adolescents’ nutritional status was classified accordingly. Additionally, for both age groups we performed the BMI z-scores according to the growth charts developed in 2000 from the Centers for Disease Control and Prevention (CDC) for the United States [34]. BMI was classified into four groups, including underweight (<−2 standard deviations, SD), normal weight (−2 to 1 SD), overweight (1 to 2 SD) and obesity (>2 SD) according to sex and age-specific cut-offs of BMI z-scores recommended by WHO in 2007. Chronic malnutrition was further defined as a z-score <−3 SD [35]. In the OKKio alla Salute survey, information on physical activity was derived from a structured questionnaire filled in by children’s parents, who provided the average number (from 0–7) of days per week the child practiced sport or played for at least 1 hour. In the HBSC survey, physical activity was provided by adolescents as the average number (from 0–7) of days per week the adolescent practiced physical exercise for at least 1 hour. Children and adolescents’ physical activity was then classified according to a tertile distribution, as low (poor physical activity, equal to fewer than 3 days/week), intermediate (3 days/week) and high (4 days/week or more).

A section of the questionnaire focused on perceived psychological wellbeing and on possible perceived threats to it: adolescents reported how often they felt low, nervous or irritable (providing a score between 1, approximately every day, to 5, rarely or never). Psychological distress was defined as the average value of the three scores. The four variables where then categorized in often, occasionally and rarely, according to the tertiles. Adolescents also reported if they had been victims of bullying in the previous two months.

### 2.2. Other Measures

In OKKio alla Salute, parents [28,31] provided information on their level of education, self-reported socio-economic status, nationality, their child’s birth weight and the average amount of time per day their child spent watching TV and played with the computer or other digital devices. Parents also provided their self-reported height and weight, that were used to derive BMI [36]. In the HBSC study [28,31], adolescents provided information on their nationality (i.e., Italian vs. not Italian), their parents’ highest level of education and their family socio-economic status, obtained as a combination of selected variables: adolescents reported if they had a family car, if they had their own bedroom, the number of computers owned by their family, if they had a dishwasher at home, the number of bathrooms at home and if they had been on any family holidays outside of Italy the year before. A total score was derived from the previous questions and adolescents’ socio-economic status was classified as low, intermediate and high, accordingly. They also reported the average amount of time per day they spent watching TV and playing with the computer or other digital devices. 

### 2.3. Statistical Analysis

The normality of the distribution was tested for anthropometric measures, and non-normally distributed variables were expressed as medians and interquartile ranges. Chi-squared tests were used to compare non-continuous variables. Assuming a sample size of 3000 subjects and an overall conservative prevalence of overweight of 14% (and alpha = 0.05), the data had sufficient statistical power (>0.80) to observe an odds ratio (OR) of 1.33, for an equally distributed dichotomous exposure variable. We used unconditional multiple logistic regression models to calculate the odds ratios (OR) and corresponding 95% confidence intervals (CI) for overweight (including obesity) and poor physical activity. All the models were adjusted for selected socio-demographic variables, i.e., sex, age (for HBSC data) and parents’ highest level of education. The ORs for overweight (including obesity) were further adjusted for physical activity (tertiles) and those for poor physical activity were adjusted for BMI (normal/underweight, overweight and obese). Additional sex-stratified analyses were performed for the ORs for overweight (including obesity), adjusting for the same variables. We also performed separate multiple logistic regression models to investigate if overweight, obesity and scant physical activity were determinants of each outcome of poor mental health: feeling low, nervous, irritable, having general psychological distress and being victims of bullying. All the models were adjusted for the same variables. A statistical weight was applied to OKKio alla Salute analyses to guarantee the representativeness of the sample at regional or LHU levels. All statistical analyses were done with software SAS, version 9.4 (Cary, NC, USA).

## 3. Results

### 3.1. BMI and Physical Activity

Table 1 shows the distribution of BMI categories and levels of physical activity among the OKKio alla Salute and HBSC study participants. Of the 3093 children aged 8–9 years, 2.3% were underweight, 75.3% normal weight, 17.6% overweight and 4.8% obese, with no significant differences between males and females (*p* = 0.109). On the total sample of 2916 adolescents aged 11–15 years, 2.9% were underweight, 83.2% normal weight, 12.2% overweight and 1.8% obese, with a higher proportion of overweight and obese adolescents among males (*p* < 0.001). A significant difference of physical activity levels was observed by gender, with a higher proportion of poorly active children and adolescents among females (*p* < 0.001). The distribution of BMI and levels of physical activity statistically significantly differed by age category (*p* = 0.001 for BMI and *p* <0.001 for physical activity). 

Supplementary Figure S1 shows the distribution of BMI levels by sex according to the z-scores. In children aged 8–9 years, the mean z-score was 0.21 (SD = 1.19) for males and 0.09 (SD = 1.10) for females. In adolescents, the mean z-score was 0.02 (SD = 1.19) for males aged 11 years, 0.12 (SD = 1.03) for those aged 13 years and −0.06 (SD = 1.15) for those aged 15 years. The corresponding values in female adolescents were −0.32 (SD = 1.08), −0.15 (SD = 1.00) and −0.19 (SD = 0.84), respectively. In adolescents, chronic malnutrition was 1.3% in males and 0.9% in females (data not shown in tables).

### 3.2. Sex, Socio-Demographic and Familial Findings

Table 2 shows the ORs and 95% CIs for childhood overweight (including obesity) and poor physical activity, according to selected socio-demographic and family characteristics, in children aged 8–9 years (OKKio alla Salute survey) and 11–15 years (HBSC survey). Among adolescents aged 11–15 years, males were more frequently overweight or obese (OR = 2.11; 95% CI: 1.69–2.64), and in both age groups they were less frequently inactive, compared to females (OR = 0.58; 95% CI: 0.50–0.68 for children aged 8–9 years; OR = 0.61; 95% CI: 0.52–0.71 for those aged 11–15 years). Poor physical activity was more frequent with increasing age (*p* for trend <0.001) and both overweight and poor physical activity were less frequent with increasing levels of parents’ education (*p* for trend from <0.001 to 0.016) in both age groups. Higher socio-economic status was related to a decreased prevalence of overweight or obesity (*p* for trend <0.001 for children aged 8–9 years; *p* for trend = 0.010 for those aged 11–15 years) and poor physical activity (*p* for trend <0.001 for adolescents aged 11–15 years). Children aged 8–9 years with a foreign nationality were less active than Italian children of the same age (OR = 1.61; 95% CI: 1.31–1.98). Overweight status was more reported with increasing child’s birth weight (*p* for trend <0.001) and in children with at least one parent obese (OR = 3.90; 95% CI: 2.98–5.11).

### 3.3. Overweight, Physical Activity and Time Spent on Screen

In both age groups, overweight was less frequent with increasing time spent for physical activity (*p* for trend <0.001 for children aged 8–9; *p* for trend = 0.001 for those aged 11–15 years; Table 3). In adolescents aged 11–15, overweight and poor physical activity were both related to greater time spent watching TV (*p* for trend <0.001) and, in both age groups, with increasing time spent playing with the computer or other digital devices (*p* for trend between 0.001 and 0.012). Poor physical activity was more frequently reported with increasing BMI (*p* for trend 0.004 for children aged 8–9 years; *p* for trend <0.001 for those aged 11–15 years).

### 3.4. Perceived Nervousness, Irritability and Experienced Bullying

In adolescents, obese subjects reported more frequently nervousness (OR = 2.37; 95% CI: 1.32–4.26), general psychological distress (OR = 2.44; 95% CI: 1.12–5.27) and bullying episodes in the previous two months (OR = 2.25; 95% CI: 1.17–4.34; Table 4). Feelings of irritability increased with an increasing level of BMI (*p* for trend = 0.038). Increasing physical activity significantly decreased the frequency of all mental health outcomes (*p* for trend ≤ 0.002).

Distribution and percent prevalence of overweight (including obese) and poor physical activity for each variable are reported in Supplementary Tables S1 and S2. Supplementary Tables S3 and S4 show the ORs and corresponding 95% CIs for childhood overweight (including obesity), stratified by sex. No substantial differences were observed.

## 4. Discussion

In 2018–2019 in northern Italy the prevalence of overweight (including obesity) was 22% in children aged 8–9 years and 14% in adolescents aged 11–15 years. The prevalence was higher in males, in families with a low socio-economic status and obese parents, and in those reporting longer time at a screen. Poor physical activity increased with age and was higher in females. Overweight and poor physical activity were both related to several psychological wellbeing shortcomings (i.e., feeling nervous, irritable) and having been a victim of bullying.

In all, the prevalence of childhood overweight in northern Italy was substantially lower than in Europe as a whole, estimated at 29% for boys and 27% for girls aged 7–9 years, and 25% for boys and 16% for girls aged 11–15 years [5,6]. Compared to the other Italian regions, Lombardy estimates showed a substantially lower prevalence of overweight [37,38], confirming a national north–south gradient [10] not only for adults [9]. Comparing our estimates with the corresponding Lombardy figures of previous waves (2014–2016), the prevalence of overweight remained substantially stable [39,40]. 

Adolescence represents a special transition period from childhood to adulthood [41], encompassing elements of psychological, biological and hormonal changes and major social role transitions, that complicate a comparison with other periods of life. However, our data, estimated using two different methods (Cole’s cut-offs and z-scores), are in line with a decrease in overweight (including obesity) prevalence from pre-pubertal age to pubertal age [5,6]. Self-reported measures in adolescents may possibly suffer reporting bias [42,43], resulting in slightly lower BMI; nevertheless, our data are consistent with those from the National Institute of Statistics, collecting data with homogeneous methodology for all age groups [8]. The already documented sharp decrease in overweight and obesity prevalence with age in Italy might be partially explained by present policies or natural course. Body dissatisfaction and the “thin-ideal of feminine beauty” are phenomena documented in adolescence, particularly in females [44,45]. These attitudes might contribute to the attenuation in overweight prevalence in pubertal age, leading to a consequent higher prevalence in malnutrition among girls. However, when comparing adolescent males and females in our data, the prevalence of chronic malnutrition was not higher among females. Among the reasons for the observed decrease in overweight prevalence with age, we can also include the possibly less healthy dietary habits of children compared to adolescents. This has been observed by a previous Italian study [46], showing a lower prevalence of good adherence to Mediterranean diet in students attending primary than secondary school, suggesting that younger children are more subjected to unhealthy choices. This result is in contrast with the majority of data from other countries reporting a negative trend in Mediterranean diet adherence with age [47,48]. In addition, the relatively high prevalence of overweight among Italian children could be related to the restricted time devoted to physical activity in the Italian primary schools compared to other countries.

Our data confirm the higher prevalence of overweight among males, and poor physical activity among females, in line with Italian and European estimates on children [5,6,49,50,51,52]. In agreement with recent European estimates, poor physical activity was more frequent with increasing age [13]. 

We confirm the well-known relationship between low socio-economic status, including parental education, and childhood overweight, obesity and poor physical activity [49]. Moreover, our estimates indicated that, particularly among children aged 8–9 years, those with a non-Italian nationality appeared at increased risk of overweight (or obesity) and poor physical activity. These results agree with other studies [53,54] and are possibly explained by the low socio-economic level of families with a foreign nationality or by the lack of knowledge among immigrant parents about available facilities and opportunities for physical activity for their children [54,55]. Therefore, preventing campaigns should focus particularly on low-socio-economic and foreign families.

Overweight and poor physical activity were more prevalent in children with at least one obese parent. These results suggest that an obese family might be a key target for intervention efforts to prevent overweight and promote adequate physical activity among these children [56,57]. In addition, as already shown [58,59], higher birth weight was a major determinant for overweight, suggesting that prenatal factors including genes and nutrition play major roles in wellness and BMI later in life.

Our findings confirmed that longer time watching TV and playing videogames was related to overweight and poor physical activity [60,61]. Parents should be aware of this direct relationship and regulate the screen time of their children. Sedentary behavior has also been associated with other unhealthy habits such as snacking on junk food [62,63]. This is particularly critical since leisure time is increasingly spent in sedentary pursuits [62,63].

In line with current literature, higher levels of BMI and poorer physical activity were related to lower psychological wellbeing, particularly in the form of feeling discomfort, such as irritability and feeling nervous [21,22,23]. In addition, adolescents who had been victims of bullying were more frequently obese and with low levels of physical activity, confirming findings from other studies highlighting the risk factors implied in such events for both psychological and physical health [24,25]. In school, weight-based bullying is among the most frequent forms of peer harassment reported by students [24,25], and weight stigmatization can further sharpen unhealthy eating behaviors and reduce physical activity [26]. Media campaigns should stress that preventing obesity has important implications not only for the physical health of children but also for their moods and psychological wellbeing. However, any dietary education and overweight prevention program, at any age group, must focus on the development of a correct relationship with food and pay particular attention to avoid the risk of developing any negative behavior, as, for example, eating disorders.

Our study needs to be interpreted in light of some limitations, mainly inherent to the cross sectional study design, not allowing us to derive any causal inference from the relationships observed. Another limitation is the self-reporting of anthropometric measures by adolescents aged 11–15 years. Moreover, the different methods of data collection used in the two surveys did not allow us to make any comparisons between the two studies. In addition, differences in sampling methodology may have caused systematic discrepancies in the two samples. Thus, for example, the proportion of foreign families was 17% among children aged 8–9 years and 4% among adolescents aged 11–15 years. In addition, since data for children and adolescents come from two different surveys (designed for different target populations), some questions were formulated differently. This may have further accentuated the observed differences between the two surveys. In addition, being this is a population-based study, we did not have the possibility to investigate any clinical data, particularly important when studying overweight correlates. New data considering clinically relevant measures are therefore required to have a clearer and more complete evaluation of correlates of overweight. Among the strengths, we can include the large sample size that enabled us to detect even the smallest differences, the representativeness of the two samples, collected using two national surveys, and the measured anthropometric data in the OKKio alla Salute survey. Another strength of this study is the comprehensiveness of the two surveys, produced in collaboration with the Regional Office for Europe of the WHO, considering exhaustively several (not frequently studied) aspects related to overweight and poor physical activity, such as psychological wellbeing and bullying, that represent the added value of this study, enabling a clearer definition of the characteristics of children and adolescents affected by overweight or obesity. In addition, limiting our cover to the Lombardy region meant we had no heterogeneous data due to differences across the country. The Lombardy region represents the most populous Italian region, with approximately 10 million inhabitants (one-sixth of Italy). Although having restricted our study to a homogeneous population is a strength of our study, in order to have a broader evaluation of the determinants of overweight, data from different countries should be collected and compared.

In conclusion, in northern Italy three out of four children or adolescents are of normal weight. Obesity intervention programs should particularly target and prioritize low socio-economic families and those with overweight or obese parents.

Special attention needs to be paid to psychological wellbeing in schools, by monitoring and possibly reducing specific threats or risk factors such as bullying or negative moods (i.e., feeling nervous and irritable). Policymakers and other stakeholders, including parents, should also increase the opportunities for young people to participate in daily physical activity and explore solutions to reduce excessive screen time in order to preserve the wellbeing of children and adolescents. Future research should evaluate the efficacy of population prevention programs focused on these identified fragile subgroups.

## Figures and Tables

**Table 1:** Percentages (%) of children from the OKKio alla Salute and HBSC surveys, according to various anthropometric measures. Lombardy, 2018–2019.

Characteristics	OKKIO alla Salute Age 8–9 Years %			HBSC Age 11–15 Years %					
	Total	Gender		Total	Gender		Age (Years)		
		Males	Females		Males	Females	11	13	15
*No. of children*	*3093*	*1595*	*1498*	*2916*	*1461*	*1455*	*859*	*1003*	*1054*
Weight (kg), median (IQR)	30.0	30.4	29.5	50.0	54.0	50.0	40.0	51.5	58.0
	(26.4–34.8)	(26.8–35.3)	(26.0–34.3)	(43.0–60.0)	(43.0–64.0)	(42.0–55.0)	(36.0–46.0)	(45.0–60.0)	(52.0–65.0)
Height (cm), median (IQR)	133.5	133.8	132.9	163.0	166.0	161.0	152.0	165.0	170.0
	(129.0–137.8)	(129.9–138.4)	(128.0–137.2)	(155.0–170.0)	(155.0–175.0)	(155.0–167.0)	(146.0–158.0)	(159.0–170.0)	(164.0–175.0)
BMI (kg/m^2^), median (IQR)	16.6	16.6	16.5	19.1	19.4	18.9	17.6	19.1	20.1
	(15.1–18.6)	(15.2–18.8)	(15.1–18.6)	(17.3–21.2)	(17.6–21.6)	(17.1–20.8)	(16.0–19.6)	(17.5–21.3)	(18.4–21.9)
BMI categories									
Underweight	2.3	1.9	2.8	2.9	2.5	3.2	4.3	1.9	2.6
Normal weight	75.3	75.1	75.5	83.2	79.2	87.2	81.6	81.9	85.7
Overweight	17.6	17.6	17.7	12.2	16.0	8.5	11.8	14.7	10.3
Obese	4.8	5.5	4.0	1.8	2.3	1.2	2.3	1.6	1.5
Physical activity (approximate tertiles) ^1^									
Low	32.9	27.2	38.9	32.8	27.6	38.0	25.1	31.8	40.0
Intermediate	29.2	31.0	27.3	19.6	17.7	21.6	19.4	20.0	19.5
High	37.9	41.7	33.8	47.5	54.7	40.4	55.5	48.2	40.5

IQR: Interquartile range. ^1^ In both surveys: <3 days per week/3 days per week/≥4 days per week. We excluded 45 children from OKKio alla Salute and 24 from HBSC because they did not provide information on physical activity.

**Table 2:** Odds ratios (OR) and corresponding 95% confidence intervals (CI) for childhood overweight (including obesity) and poor physical activity according to selected socio-demographic and family characteristics. Lombardy, 2018–2019.

Characteristics	Overweight ^1^		Poor Physical Activity ^2^	
	OR (95% CI)		OR (95% CI)	
	OKKio alla Salute Age 8–9 Years	HBSC Age 11–15 Years	OKKio alla Salute Age 8–9 Years	HBSC Age 11–15 Years
***Total, No.***	*3021*	*2833*	*3048*	*2892*
Sex				
Female	1.00 ^3^	1.00 ^3^	1.00 ^3^	1.00 ^3^
Male	1.13 (0.95–1.34)	**2.11 (1.69–2.64)**	**0.58 (0.50–0.68)**	**0.61 (0.52–0.71)**
Age category				
11	-	1.00°	-	1.00°
13	-	1.14 (0.87–1.51)	-	**1.52 (1.22–1.89)**
15	-	0.79 (0.58–1.06)	-	**2.18 (1.75–2.71)**
*p* for trend	-	0.079	-	**<0.001**
Highest parental education				
Low	1.00 ^3^	1.00 ^3^	1.00 ^3^	1.00 ^3^
Intermediate	**0.71 (0.57–0.90)**	0.76 (0.49–1.17)	**0.78 (0.63–0.96)**	0.73 (0.52–1.01)
High	**0.55 (0.43–0.70)**	**0.58 (0.37–0.91)**	**0.74 (0.59–0.92)**	**0.58 (0.41–0.82)**
*p* for trend	**<0.001**	**0.009**	**0.016**	**0.001**
Family socio-economic status				
Low	1.00 ^3^	1.00 ^3^	1.00 ^3^	1.00 ^3^
Intermediate	**0.71 (0.59–0.86)**	0.87 (0.67–1.14)	**0.79 (0.67–0.95)**	**0.72 (0.59–0.88)**
High	**0.55 (0.41–0.73)**	**0.65 (0.47–0.90)**	1.02 (0.80–1.30)	**0.63 (0.50–0.80)**
*p* for trend	**<0.001**	**0.010**	0.482	**<0.001**
Nationality				
Italian	1.00 ^3^	1.00 ^3^	1.00 ^3^	1.00 ^3^
Other	1.23 (0.98–1.54)	1.31 (0.80–2.14)	**1.61 (1.31–1.98)**	0.87 (0.58–1.32)
Birth weight (g) ^4^				
<2500	1.00 ^3^	-	1.00 ^3^	-
2500–3300	1.08 (0.75–1.56)	-	0.83 (0.63–1.11)	-
>3300	**1.72 (1.20–2.47)**	-	0.81 (0.61–1.09)	-
*p* for trend	**<0.001**	-	0.267	-
Parental BMI ^4^				
Both parents normal weight	1.00 ^3^	-	1.00 ^3^	-
At least one parent overweight (not obese)	**1.95 (1.56–2.44)**	-	1.08 (0.90–1.29)	-
At least one parent obese	**3.90 (2.98–5.11)**	-	1.06 (0.83–1.37)	-
*p* for trend	**<0.001**	-	0.508	-

^1^ ORs for overweight (overweight/obesity vs. normal weight) were calculated in unconditional multiple logistic regression models, after adjustment for sex, parents’ highest level of education and physical activity (low, intermediate, high); HBSC data were further adjusted for age. Underweight children and adolescents were excluded from the analyses. Estimates in bold type are significant at 0.05. ^2^ ORs for poor physical activity (first vs. second and third tertiles of physical activity) were calculated in unconditional multiple logistic regression models, after adjustment for sex, parents’ highest level of education and BMI (underweight/normal weight, overweight, obese); HBSC data were further adjusted for age; 45 children from OKKio alla Salute and 24 from HBSC did not provide information on physical activity and were therefore excluded. Estimates in bold type are significant at 0.05. ^3^ Reference category. ^4^ Information on the child’s birth weight and parents’ BMI were not available for the HBSC survey.

**Table 3:** Odds ratios (OR) and corresponding 95% confidence intervals (CI) for childhood overweight (including obesity) and poor physical activity according to selected lifestyle habits and other characteristics. Lombardy, 2018–2019.

Characteristics	Overweight ^1^		Poor Physical Activity ^2^	
	OR (95% CI)		OR (95% CI)	
	OKKio alla Salute Age 8–9 Years	HBSC Age 11–15 Years	OKKio alla Salute Age 8–9 Years	HBSC Age 11–15 Years
Physical activity				
Low	1.00 ^3^	1.00 ^3^	-	-
Moderate	0.82 (0.66–1.02)	0.85 (0.63–1.14)	-	-
High	**0.67 (0.54–0.82)**	**0.66 (0.52–0.85)**	-	-
*p* for trend	**<0.001**	**0.001**	-	-
Time spent watching TV (approximate tertiles) ^4^				
Low	1.00 ^3^	1.00 ^3^	1.00 ^3^	1.00 ^3^
Intermediate	1.11 (0.87–1.41)	1.09 (0.81–1.46)	0.90 (0.72–1.12)	0.90 (0.73–1.11)
High	1.18 (0.95–1.46)	**1.81 (1.40–2.33)**	1.19 (0.99–1.44)	**1.44 (1.19–1.74)**
*p* for trend	0.132	**<0.001**	0.091	**<0.001**
Time spent playing with the computer (approximate tertiles) ^5^				
Low	1.00 ^3^	1.00 ^3^	1.00 ^3^	1.00 ^3^
Intermediate	1.14 (0.90–1.45)	1.26 (0.93–1.71)	1.14 (0.93–1.40)	1.18 (0.95–1.45)
High	**1.46 (1.15–1.85)**	**1.65 (1.22–2.22)**	**1.37 (1.10–1.69)**	**1.31 (1.06–1.62)**
*p* for trend	**0.002**	**0.001**	**0.004**	**0.012**
BMI				
Underweight	-	-	**1.65 (1.01–2.70)**	1.49 (0.94–2.36)
Normal weight	-	-	1.00 ^3^	1.00 ^3^
Overweight	-	-	**1.28 (1.05–1.57)**	**1.29 (1.02–1.65)**
Obese	-	-	**1.71 (1.21–2.42)**	**2.33 (1.32–4.13)**
*p* for trend	-	-	**0.004**	**<0.001**

^1^ ORs for overweight (overweight/obesity vs. normal weight) were calculated in unconditional multiple logistic regression models, after adjustment for sex, parents’ highest level of education and physical activity (low, intermediate, high). HBSC data were further adjusted for age. Underweight children and adolescents were excluded from the analyses. Estimates in bold type are significant at 0.05. ^2^ ORs for poor physical activity (first vs. second and third tertiles of physical activity) were calculated in unconditional multiple logistic regression models, after adjustment for sex, parents’ highest level of education and BMI (underweight/normal weight, overweight, obese); HBSC data were further adjusted for age; 45 children from OKKio alla Salute and 24 from HBSC did not provide information on physical activity and were therefore excluded. Estimates in bold type are significant at 0.05. ^3^ Reference category. ^4^ In OKKio alla Salute: <1.17 h per day/1.17–1.54 h/≥1.55 h per day. In HBSC: <1.17 h per day/1.17–2.22 h/≥2.23 h per day. ^5^ In OKKio alla Salute: <34 min per day /34 min–1.16 h/≥1.17 h per day. In HBSC: <38 min per day/38 min −1.51 h/≥1.52 h per day.

**Table 4:** Odds ratios ^1^ (OR) and corresponding 95% confidence intervals (CI) for selected mental health outcomes by body mass index (BMI) levels and physical activity. Lombardy, 2018.

Characteristics	HBSC Age 11–15 Years				
	Nervous	Feeling Low	Irritable	Psychological Distress ^2^	Being Bullied
	OR (95% CI)	OR (95% CI)	OR (95% CI)	OR (95% CI)	OR (95% CI)
BMI levels					
Underweight	0.89 (0.56–1.40)	1.16 (0.73–1.83)	0.83 (0.53–1.31)	0.97 (0.60–1.59)	1.42 (0.79–2.53)
Normal weight	1.00 ^3^	1.00 ^3^	1.00 ^3^	1.00 ^3^	1.00 ^3^
Overweight	1.15 (0.91–1.45)	1.18 (0.93–1.49)	1.20 (0.95–1.52)	1.09 (0.84–1.40)	1.25 (0.91–1.72)
Obese	**2.37 (1.32–4.26)**	1.36 (0.76–2.45)	1.44 (0.79–2.62)	**2.44 (1.12–5.27)**	**2.25 (1.17–4.34)**
*p* for trend	**0.007**	0.203	**0.038**	0.075	0.069
Physical activity					
Low	1.00 ^3^	1.00 ^3^	1.00 ^3^	1.00 ^3^	1.00 ^3^
Moderate	**0.66 (0.53–0.81)**	**0.76 (0.61–0.95)**	**0.77 (0.62–0.96)**	**0.66 (0.52–0.84)**	**0.67 (0.49–0.93)**
High	**0.74 (0.62–0.88)**	**0.75 (0.63–0.89)**	**0.67 (0.56–0.80)**	**0.62 (0.51–0.75)**	**0.72 (0.56–0.92)**
*p* for trend	**<0.001**	**0.002**	**<0.001**	**<0.001**	**0.001**

^1^ ORs for all mental health outcomes were calculated in unconditional multiple logistic regression models, after adjustment for sex, age group, parents’ highest level of education and mutually for BMI levels and physical activity. Estimates in bold type are significant at 0.05. ^2^ Psychological distress was computed as the average score of the three previous measures (nervousness, feeling low, irritability). ^3^ Reference category.

## Data Availability

The data presented in this study are available on request from the corresponding author.

## Supplementary Materials

The following supporting information can be downloaded at: https://www.mdpi.com/article/10.3390/nu14112258/s1. Figure S1: Distribution of children aged 8–9 years and adolescents aged 11–15 years according to BMI levels measured with z-scores. Table S1: Frequencies and percent distribution (%) of overweight (including obesity) and poor physical activity according to selected socio-demographic and family characteristics. Lombardy, 2018–2019. Table S2: Frequencies and percent distribution (%) of overweight (including obesity) and poor physical activity according to selected lifestyle habits and other characteristics. Lombardy, 2018–2019. Table S3: Odds ratios (OR) and corresponding 95% confidence intervals (CI) for childhood overweight (including obesity) stratified by sex, according to selected socio-demographic and family characteristics. Lombardy, 2018–2019. Table S4: Odds ratios (OR) and corresponding 95% confidence intervals (CI) for childhood overweight (including obesity) stratified by sex, according to selected lifestyle habits and other characteristics. Lombardy, 2018–2019.

## References

[1] Garrido-Miguel M., Martínez-Vizcaíno V., Oliveira A., Martínez-Andrés M., Sequí-Domínguez I., Hernández-Castillejo L.E., Cavero-Redondo I. (2021). Prevalence and trends of underweight in European children and adolescents: A systematic review and meta-analysis. Eur. J. Nutr..

[2] Swinburn B.A., Sacks G., Hall K.D., McPherson K., Finegood D.T., Moodie M.L., Gortmaker S.L. (2011). The global obesity pandemic: Shaped by global drivers and local environments. Lancet.

[3] NCD Risk Factor Collaboration (2017). Worldwide trends in body-mass index, underweight, overweight, and obesity from 1975 to 2016: A pooled analysis of 2416 population-based measurement studies in 128.9 million children, adolescents, and adults. Lancet.

[4] Garrido-Miguel M., Cavero-Redondo I., Álvarez-Bueno C., Rodríguez-Artalejo F., Moreno L.A., Ruiz J.R., Ahrens W., Martínez-Vizcaíno V. (2019). Prevalence and Trends of Overweight and Obesity in European Children From 1999 to 2016: A Systematic Review and Meta-analysis. JAMA Pediatr..

[5] World Health Organization (WHO) WHO European Childhood Obesity Surveillance Initiative (COSI). Report on the Fourth Round of Data Collection, 2015–2017. https://www.euro.who.int/en/health-topics/noncommunicable-diseases/obesity/publications/2021/who-european-childhood-obesity-surveillance-initiative-cosi-report-on-the-fourth-round-of-data-collection,-20152017-2021.

[6] World Health Organization (WHO) Spotlight on Adolescent Health and Well-Being. Findings from the 2017/2018 Health Behaviour in School-Aged Children (HBSC) Survey in Europe and Canada. International Report. Volume 1. Key Findings. https://www.euro.who.int/en/health-topics/Life-stages/child-and-adolescent-health/health-behaviour-in-school-aged-children-hbsc/publications/2020/spotlight-on-adolescent-health-and-well-being.-findings-from-the-20172018-health-behaviour-in-school-aged-children-hbsc-survey-in-europe-and-canada.-international-report.-volume-1.-key-findings.

[7] Eurostat (2019). Overweight and Obesity—BMI Statistics. https://ec.europa.eu/eurostat/statistics-explained/index.php?title=Overweight_and_obesity_-_BMI_statistics#Obesity_by_age_group.

[8] ISTAT Stili Di Vita Di Bambini E Ragazzi. Anni 2017–2018. https://www.istat.it/it/archivio/234930.

[9] Lazzeri G., Giacchi M.V., Spinelli A., Pammolli A., Dalmasso P., Nardone P., Lamberti A., Cavallo F. (2014). Overweight among students aged 11–15 years and its relationship with breakfast, area of residence and parents’ education: Results from the Italian HBSC 2010 cross-sectional study. Nutr. J..

[10] Gallus S., Odone A., Lugo A., Bosetti C., Colombo P., Zuccaro P., La Vecchia C. (2013). Overweight and obesity prevalence and determinants in Italy: An update to 2010. Eur. J. Nutr..

[11] Guthold R., Stevens G.A., Riley L.M., Bull F.C. (2018). Worldwide trends in insufficient physical activity from 2001 to 2016: A pooled analysis of 358 population-based surveys with 1.9 million participants. Lancet Glob. Health.

[12] Musić Milanović S., Buoncristiano M., Križan H., Rathmes G., Williams J., Hyska J., Duleva V., Zamrazilová H., Hejgaard T., Jørgensen M.B. (2021). Socioeconomic disparities in physical activity, sedentary behavior and sleep patterns among 6- to 9-year-old children from 24 countries in the WHO European region. Obes. Rev..

[13] Schwarzfischer P., Gruszfeld D., Stolarczyk A., Ferre N., Escribano J., Rousseaux D., Moretti M., Mariani B., Verduci E., Koletzko B. (2019). Physical Activity and Sedentary Behavior From 6 to 11 Years. Pediatrics.

[14] Guthold R., Stevens G.A., Riley L.M., Bull F.C. (2020). Global trends in insufficient physical activity among adolescents: A pooled analysis of 298 population-based surveys with 1.6 million participants. Lancet Child Adolesc. Health.

[15] Marzi I., Tcymbal A., Gelius P., Abu-Omar K., Reimers A.K., Whiting S., Wickramasinghe K. (2021). Monitoring of physical activity promotion in children and adolescents in the EU: Current status and future perspectives. Eur. J. Public Health.

[16] World Health Organization (WHO) Physical Actvity Country Fact Sheets. https://www.euro.who.int/en/health-topics/disease-prevention/physical-activity/data-and-statistics/physical-activity-fact-sheets/physical-activity-country-factsheets.

[17] Konstabel K., Veidebaum T., Verbestel V., Moreno L.A., Bammann K., Tornaritis M., Eiben G., Molnár D., Siani A., Sprengeler O. (2014). Objectively measured physical activity in European children: The IDEFICS study. Int. J. Obes..

[18] Spitzer M. (2021). Open schools! Weighing the effects of viruses and lockdowns on children. Trends Neurosci. Educ..

[19] Sutaria S., Devakumar D., Yasuda S.S., Das S., Saxena S. (2019). Is obesity associated with depression in children? Systematic review and meta-analysis. Arch. Dis. Child..

[20] Goran M.I., Treuth M.S. (2001). Energy expenditure, physical activity, and obesity in children. Pediatr. Clin. N. Am..

[21] Korczak D.J., Madigan S., Colasanto M. (2017). Children’s Physical Activity and Depression: A Meta-analysis. Pediatrics.

[22] Rothon C., Edwards P., Bhui K., Viner R.M., Taylor S., Stansfeld S.A. (2010). Physical activity and depressive symptoms in adolescents: A prospective study. BMC Med..

[23] Biddle S.J., Asare M. (2011). Physical activity and mental health in children and adolescents: A review of reviews. Br. J. Sports Med..

[24] Pont S.J., Puhl R., Cook S.R., Slusser W., Section on Obesity, The Obesity Society (2017). Stigma Experienced by Children and Adolescents with Obesity. Pediatrics.

[25] Puhl R., Suh Y. (2015). Health Consequences of Weight Stigma: Implications for Obesity Prevention and Treatment. Curr. Obes. Rep..

[26] Rao W.W., Zong Q.-Q., Zhang J.-W., An F.-R., Jackson T., Ungvari G.S., Xiang Y., Su Y.-Y., D’Arcy C., Xiang Y. (2020). Obesity increases the risk of depression in children and adolescents: Results from a systematic review and meta-analysis. J. Affect. Disord..

[27] ISTAT Popolazione Residente—Bilancio: Lombardia. http://dati.istat.it/index.aspx?queryid=18968.

[28] Spinelli A., Lamberti A., Baglio G., Andreozzi S., Galeone D.E., Sanità I.S.D. (2009). OKkio alla SALUTE: Sistema di Sorveglianza su Alimentazione e Attività Fisica Nei Bambini della Scuola Primaria.

[29] Inchley J.C.D., Cosma A., Samdal O. (2018). Health Behaviour in School-Aged Children (HBSC) Study Protocol: Background, Methodology and Mandatory Items for the 2017/18 Survey.

[30] Varghese N.E., Santoro E., Lugo A., Madrid-Valero J.J., Ghislandi S., Torbica A., Gallus S. (2021). The Role of Technology and Social Media Use in Sleep-Onset Difficulties Among Italian Adolescents: Cross-sectional Study. J. Med. Internet Res..

[31] Currie C.I.J., Molcho M., Lenzi M., Veselska Z., Wild F. (2014). Health Behaviour in School-Aged Children (HBSC) Study Protocol: Background, Methodology and Mandatory Items for the 2013/14 Survey.

[32] Cole T.J., Bellizzi M.C., Flegal K.M., Dietz W.H. (2000). Establishing a standard definition for child overweight and obesity worldwide: International survey. BMJ.

[33] Cole T.J., Flegal K.M., Nicholls D., Jackson A.A. (2007). Body mass index cut offs to define thinness in children and adolescents: International survey. BMJ.

[34] Kuczmarski R.J., Ogden C.L., Guo S.S., Grummer-Strawn L.M., Flegal K.M., Mei Z., Wei R., Curtin L.R., Roche A.F., Johnson C.L. (2002). 2000 CDC Growth Charts for the United States: Methods and development. Vital Health Stat..

[35] Li Y., Di Gao D., Liu J., Yang Z., Wen B., Chen L., Chen M., Ma Y., Ma T., Bin Dong B. (2022). Prepubertal BMI, pubertal growth patterns, and long-term BMI: Results from a longitudinal analysis in Chinese children and adolescents from 2005 to 2016. Eur. J. Clin. Nutr..

[36] World Health Organization (WHO) (2000). Obesity: Preventing and Managing the Global Epidemic.

[37] Istituto Superiore di Sanità (ISS) Indagine Nazionale 2019: I Dati Nazionali. https://www.epicentro.iss.it/okkioallasalute/indagine-2019-dati.

[38] Istituto Superiore di Sanità (ISS) Report HBSC 2018. https://www.epicentro.iss.it/hbsc/indagine-2018.

[39] Istituto Superiore di Sanità (ISS) Indagine 2016: Dati Regionali. https://www.epicentro.iss.it/okkioallasalute/ReportRegionali2016.

[40] Istituto Superiore di Sanità (ISS) Report HBSC 2014. https://www.epicentro.iss.it/hbsc/indagine-2014.

[41] Sawyer S.M., Azzopardi P.S., Wickremarathne D., Patton G.C. (2018). The age of adolescence. Lancet Child Adolesc. Health.

[42] Bostrom G., Diderichsen F. (1997). Socioeconomic differentials in misclassification of height, weight and body mass index based on questionnaire data. Int. J. Epidemiol..

[43] Lin C.J., DeRoo L.A., Jacobs S.R., Sandler D.P. (2012). Accuracy and reliability of self-reported weight and height in the Sister Study. Public Health Nutr..

[44] Rodgers R., Chabrol H. (2009). The impact of exposure to images of ideally thin models on body dissatisfaction in young French and Italian women. Encephale.

[45] Verri A.P., Verticale M.S., Vallero E., Bellone S., Nespoli L. (1997). Television and eating disorders. Study of adolescent eating behavior. Minerva Pediatr..

[46] Archero F., Ricotti R., Solito A., Carrera D., Civello F., Di Bella R., Bellone S., Prodam F. (2018). Adherence to the Mediterranean Diet among School Children and Adolescents Living in Northern Italy and Unhealthy Food Behaviors Associated to Overweight. Nutrients.

[47] Iaccarino Idelson P., Scalfi L., Valerio G. (2017). Adherence to the Mediterranean Diet in children and adolescents: A systematic review. Nutr. Metab. Cardiovasc. Dis..

[48] Grosso G., Galvano F. (2016). Mediterranean diet adherence in children and adolescents in southern European countries. NFS J..

[49] Lazzeri G., Dalmasso P., Berchialla P., Borraccino A., Charrier L., Giacchi M.V., Simi R., Lenzi M., Vieno A., Lemma P. (2017). Trends in adolescent overweight prevalence in Italy according to socioeconomic position. Ann. Ist. Super Sanità.

[50] Lauria L., Spinelli A., Buoncristiano M., Nardone P. (2019). Decline of childhood overweight and obesity in Italy from 2008 to 2016: Results from 5 rounds of the population-based surveillance system. BMC Public Health.

[51] Haug E., Rasmussen M., Samdal O., Iannotti R., Kelly C., Borraccino A., Vereecken C., Melkevik O., Lazzeri G., Giacchi M. (2009). Overweight in school-aged children and its relationship with demographic and lifestyle factors: Results from the WHO-Collaborative Health Behaviour in School-aged Children (HBSC) study. Int. J. Public Health.

[52] Whiting S., Buoncristiano M., Gelius P., Abu-Omar K., Pattison M., Hyska J., Duleva V., Milanović S.M., Zamrazilová H., Hejgaard T. (2021). Physical Activity, Screen Time, and Sleep Duration of Children Aged 6–9 Years in 25 Countries: An Analysis within the WHO European Childhood Obesity Surveillance Initiative (COSI) 2015–2017. Obes. Facts.

[53] Gualdi-Russo E., Zaccagni L., Manzon V.S., Masotti S., Rinaldo N., Khyatti M. (2014). Obesity and physical activity in children of immigrants. Eur. J. Public Health.

[54] Besharat Pour M., Bergström A., Bottai M., Kull I., Wickman M., Håkansson N., Wolk A., Moradi T. (2014). Effect of parental migration background on childhood nutrition, physical activity, and body mass index. J. Obes..

[55] Van Hook J., Baker E., Altman C.E., Frisco M.L. (2012). Canaries in a coalmine: Immigration and overweight among Mexican-origin children in the US and Mexico. Soc. Sci. Med..

[56] Sigmund E., Sigmundova D., Badura P. (2020). Excessive body weight of children and adolescents in the spotlight of their parents’ overweight and obesity, physical activity, and screen time. Int. J. Public Health.

[57] Wang Y., Min J., Khuri J., Li M. (2017). A Systematic Examination of the Association between Parental and Child Obesity across Countries. Adv. Nutr..

[58] Rito A.I., Buoncristiano M., Spinelli A., Salanave B., Kunešová M., Hejgaard T., Solano M.G., Fijałkowska A., Sturua L., Hyska J. (2019). Association between Characteristics at Birth, Breastfeeding and Obesity in 22 Countries: The WHO European Childhood Obesity Surveillance Initiative—COSI 2015/2017. Obes. Facts.

[59] Yuan Z.P., Yang M., Liang L., Fu J.F., Xiong F., Liu G.L., Gong C.X., Luo F.H., Chen S.K., Zhang D.D. (2015). Possible role of birth weight on general and central obesity in Chinese children and adolescents: A cross-sectional study. Ann. Epidemiol..

[60] Mineshita Y., Kim H.K., Chijiki H., Nanba T., Shinto T., Furuhashi S., Oneda S., Kuwahara M., Suwama A., Shibata S. (2021). Screen time duration and timing: Effects on obesity, physical activity, dry eyes, and learning ability in elementary school children. BMC Public Health.

[61] Robinson T.N., Banda J.A., Hale L., Lu A.S., Fleming-Milici F., Calvert S.L., Wartella E. (2017). Screen Media Exposure and Obesity in Children and Adolescents. Pediatrics.

[62] Biddle S.J., Petrolini I., Pearson N. (2014). Interventions designed to reduce sedentary behaviours in young people: A review of reviews. Br. J. Sports Med..

[63] Belton S., Issartel J., Behan S., Goss H., Peers C. (2021). The Differential Impact of Screen Time on Children’s Wellbeing. Int. J. Environ. Res. Public Health.

