# Supplementary material for: Prevalence and Correlates of Overweight, Obesity and Physical Activity in Italian Children and Adolescents from Lombardy, Italy
Source: Nutrients. 2022 May 28;14(11):2258. doi: 10.3390/nu14112258 (PMC9182936; doi:10.3390/nu14112258)
Supplement: Supplementary file 1 [file nutrients-14-02258-s001.zip › Supplementary Table S1.pdf]

Supplementary Table S1. Frequencies and percent distribution (%) of overweight (including obesity) and poor physical activity according to selected socio-demographic and family characteristics. Lombardy, 2018-2019.

Characteristics	OKKio alla Salute Age 8-9 years			HBSC Age 11-15 years		
	No.	Overweight %	Poor physical activity[1] %	No.	Overweight %	Poor physical activity[1] %
Total, No. (%)	*3,093*	*22.4*	*32.9*	*2,916*	*14.0*	*32.8*
Sex						
Females	1498	21.7	38.9	1455	9.7	38.0
Males	1595	23.0	27.2	1461	18.3	27.6
Age category						
11	-	-	-	859	14.1	25.1
13	-	-	-	1003	16.3	31.8
15	-	-	-	1054	11.8	40.0
Highest parental education						
Low	504	29.6	38.4	175	18.3	42.4
Intermediate	1422	22.4	32.1	1160	14.2	34.5
High	1046	18.3	30.2	866	11.0	28.2
Family socio-economic status						
Low	1086	27.7	35.7	614	17.9	40.8
Intermediate	1375	20.5	29.4	1412	14.5	32.1
High	452	15.7	34.8	827	10.4	28.2
Nationality						
Italian	2474	21.0	30.5	2775	13.8	32.8
Other	503	28.1	42.8	118	19.5	31.3
Birth weight (gr) [2]						
<2500	249	16.7	36.6	-	-	-
2500-3300	1426	19.0	32.8	-	-	-
>3300	1265	27.5	31.8	-	-	-
Parental BMI [2]						
Both parents normal weight	1074	12.9	29.9	-	-	-
At least one parent overweight (not obese)	1250	23.2	32.5	-	-	-
At least one parent obese	423	38.9	34.2	-	-	-

[1] We excluded 45 children from OKKio alla Salute and 24 from HBSC because they did not provide information on physical activity.
[2] Information on baby's birth weight and parents' BMI were not available for the HBSC survey.
