# Supplementary material for: Prevalence and Correlates of Overweight, Obesity and Physical Activity in Italian Children and Adolescents from Lombardy, Italy
Source: Nutrients. 2022 May 28;14(11):2258. doi: 10.3390/nu14112258 (PMC9182936; doi:10.3390/nu14112258)
Supplement: Supplementary file 1 [file nutrients-14-02258-s001.zip › Supplementary Table S2.pdf]

Supplementary Table S2. Frequencies and percent distribution (%) of overweight (including obesity) and poor physical activity according to selected lifestyle habits and other characteristics. Lombardy, 2018-2019.

Characteristics	OKKio alla Salute Age 8-9 years			HBSC Age 11-15 years		
	No.	Overweight %	Poor physical activity[1] %	No.	Overweight %	Poor physical activity[1] %
Physical activity (Days/week)[2]						
Low	1002	26.2	-	949	15.8	-
Moderate	891	22.4	-	568	14.4	-
High	1155	19.2	-	1375	12.4	-
Time spent watching TV (approximate tertiles)[3]						
Low	1193	20.3	31.3	1129	11.2	29.7
Intermediate	593	22.3	29.1	752	12.1	28.3
High	887	24.6	35.9	969	18.9	39.5
Time spent playing with the computer (approximate tertiles)[4]						
Low	859	18.8	30.4	932	9.1	33.0
Intermediate	945	21.5	31.3	961	13.7	30.8
High	873	27.1	34.7	969	19.0	34.2
BMI						
Thin	72	-	43.8	83	-	39.8
Normal weight	2329	-	30.9	2424	-	31.8
Overweight	545	-	36.9	356	-	35.5
Obese	147	-	43.8	52	-	49.0

[1] We excluded 45 children from OKKio alla Salute and 24 from HBSC because they did not provide information on physical activity.

[2] In both surveys:<3 days per week/3 days per week /≥4 days per week.

[3] In OKKio alla Salute: <1.17 hours per day/1.17 -1.54 hours/≥ 1.55 hours per day. In HBSC: <1.17 hours per day/1.17 -2.22 hours/≥2.23 hours per day.

[4] In OKKio alla Salute: <34 minutes per day /34 minutes-1.16 hours /≥ 1.17 hours per day. In HBSC: <38 minutes per day/38 minutes -1.51 hours/≥1.52 hours per day.
