# Supplementary material for: Prevalence and Correlates of Overweight, Obesity and Physical Activity in Italian Children and Adolescents from Lombardy, Italy
Source: Nutrients. 2022 May 28;14(11):2258. doi: 10.3390/nu14112258 (PMC9182936; doi:10.3390/nu14112258)
Supplement: Supplementary file 1 [file nutrients-14-02258-s001.zip › Supplementary Table S3.pdf]

Supplementary Table S3. Odds ratios (OR) and corresponding 95% confidence intervals (CI) for childhood overweight (including obesity) stratified by sex, according to selected socio-demographic and family characteristics. Lombardy, 2018-2019.

Characteristics	Overweight[1] OR (95% CI)			
	OKKio alla Salute Age 8-9 years		HBSC Age 11-15 years	
	Males	Females	Males	Females
Total	*1565*	*1456*	*1424*	*1409*
Age category				
11	-	-	1.00[2]	1.00[2]
13	-	-	1.23 (0.87-1.74)	0.99 (0.62-1.57)
15	-	-	0.84 (0.58-1.24)	0.67 (0.41-1.09)
P for trend			0.327	0.075
Highest parental education				
Low	1.00[2]	1.00[2]	1.00[2]	1.00[2]
Intermediate	**0.68 (0.49-0.95)**	0.75 (0.54-1.04)	0.71 (0.41-1.23)	0.81 (0.41-1.61)
High	**0.63 (0.45-0.89)**	**0.46 (0.31-0.66)**	0.57 (0.32-1.02)	0.57 (0.27-1.19)
P for trend	**0.016**	**<0.001**	0.052	0.083
Family socio-economic status				
Low	1.00[2]	1.00[2]	1.00[2]	1.00[2]
Middle	**0.73 (0.56-0.95)**	**0.69 (0.52-0.91)**	0.96 (0.68-1.35)	0.73 (0.48-1.11)
High	**0.40 (0.25-0.63)**	0.71 (0.47-1.05)	0.80 (0.53-1.21)	**0.46 (0.26-0.79)**
P for trend	**<0.001**	**0.022**	0.282	**0.005**
Nationality				
Italian	1.00[2]	1.00[2]	1.00[2]	1.00[2]
Other	**1.43 (1.05-1.94)**	1.02 (0.72-1.44)	1.08 (0.60-1.96)	1.98 (0.86-4.59)
Birth weight (gr) [3]				
<2500	1.00[2]	1.00[2]		
2500-3300	**2.06 (1.08-3.94)**	0.74 (0.47-1.16)		
>3300	**3.31 (1.74-6.29)**	1.14 (0.72-1.81)		
P for trend	**<0.001**	**0.040**		
Parental BMI [3]				
Both parents normal weight	1.00[2]	1.00[2]		
At least one parent overweight (not obese)	**2.23 (1.64-3.03)**	**1.72 (1.23-2.39)**		
At least one parent obese	**3.73 (2.56-5.44)**	**4.15 (2.81-6.14)**		
P for trend	**<0.001**	**<0.001**		

[1] ORs for overweight (overweight/obesity vs. normal weight) were calculated in unconditional multiple logistic regression models, after adjustment for sex, parents' highest level of education and physical activity (low, intermediate, high); HBSC data were further adjusted for age. Thin children and adolescents were excluded from the analyses. Estimates in bold type are significant at 0.05.

[2] Reference category.

[3] Information on baby's birth weight and parents' BMI were not available for the HBSC survey.
