# Supplementary material for: Prevalence and Correlates of Overweight, Obesity and Physical Activity in Italian Children and Adolescents from Lombardy, Italy
Source: Nutrients. 2022 May 28;14(11):2258. doi: 10.3390/nu14112258 (PMC9182936; doi:10.3390/nu14112258)
Supplement: Supplementary file 1 [file nutrients-14-02258-s001.zip › Supplementary Table S4.pdf]

Supplementary Table S4. Odds ratios (OR) and corresponding 95% confidence intervals (CI) for childhood overweight (including obesity) stratified by sex, according to selected lifestyle habits and other characteristics. Lombardy, 2018-2019.

Characteristics	Overweight[1] OR (95% CI)			
	OKKio alla Salute		HBSC	
	Males	Females	Males	Females
Physical activity				
Low	1.00[2]	1.00[2]	1.00[2]	1.00[2]
Intermediate	0.85 (0.63-1.15)	0.79 (0.58-1.08)	0.80 (0.54-1.17)	0.91 (0.57-1.47)
High	**0.69 (0.52-0.92)**	**0.65 (0.48-0.87)**	**0.55 (0.40-0.75)**	0.92 (0.62-1.37)
P for trend	**0.010**	**0.004**	**<0.001**	0.672
Time spent watching TV (approximate tertiles) [3]				
Low	1.00[2]	1.00[2]	1.00[2]	1.00[2]
Intermediate	1.17 (0.84-1.63)	1.03 (0.72-1.47)	1.24 (0.86-1.80)	0.93 (0.57-1.51)
High	1.16 (0.86-1.56)	1.20 (0.88-1.63)	**2.04 (1.46-2.83)**	**1.56 (1.04-2.33)**
P for trend	0.314	0.249	**<0.001**	**0.031**
Time spent playing with the computer (approximate tertiles)[4]				
Low	1.00[2]	1.00[2]	1.00[2]	1.00[2]
Intermediate	**1.50 (1.04-2.15)**	0.93 (0.67-1.29)	**1.88 (1.12-3.15)**	1.08 (0.71-1.66)
High	**1.87 (1.31-2.68)**	1.24 (0.89-1.74)	**2.66 (1.61-4.40)**	1.13 (0.72-1.76)
P for trend	**0.001**	0.247	**<0.001**	0.578

[1] ORs for overweight (overweight/obesity vs. normal weight) were calculated in unconditional multiple logistic regression models, after adjustment for sex, parents' highest level of education and physical activity (low, intermediate, high). HBSC data were further adjusted for age. Thin children and adolescents were excluded from the analyses. Estimates in bold type are significant at 0.05.

[2] Reference category.

[3] In OKKio alla Salute: <1.17 hours per day/1.17 -1.54 hours/≥ 1.55 hours per day. In HBSC: <1.17 hours per day/1.17 -2.22 hours/≥2.23 hours per day.

[4] In OKKio alla Salute: <34 minutes per day /34 minutes-1.16 hours /≥ 1.17 hours per day. In HBSC: <38 minutes per day/38 minutes -1.51 hours/≥1.52 hours per day.
