# Supplementary figures and images for: Prevalence and Correlates of Overweight, Obesity and Physical Activity in Italian Children and Adolescents from Lombardy, Italy
Source: Nutrients. 2022 May 28;14(11):2258. doi: 10.3390/nu14112258 (PMC9182936; doi:10.3390/nu14112258)

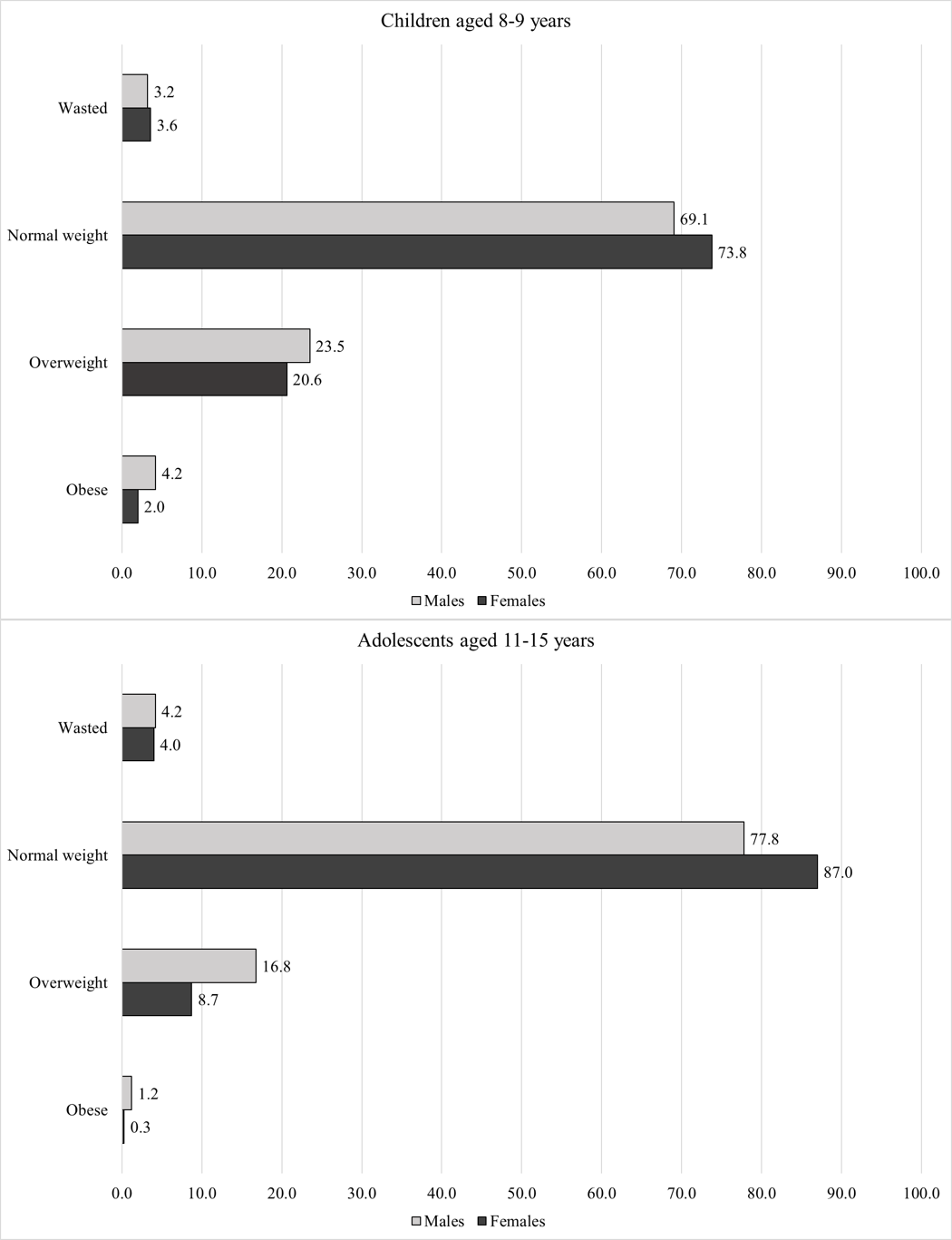

Supplement: Supplementary file 1 [file nutrients-14-02258-s001.zip › Figure S1.png]
